# A molecular study on recombinant pullulanase type I from *Metabacillus* *indicus*

**DOI:** 10.1186/s13568-023-01545-8

**Published:** 2023-04-29

**Authors:** Zahraa Z. Al-Mamoori, Amira M. Embaby, Ahmed Hussein, Hoda E. Mahmoud

**Affiliations:** Biotechnology Department, Institute of Graduate Studies and Research, Alexandria University, Alexandria, Egypt

**Keywords:** Cold-adapted pullulanase type I, *Metabacillus indicus*, Heterologous expression, Biochemical characterization, *In silico* structural modeling

## Abstract

Despite the great potential of cold-adapted pullulanase type I in tremendous industrial applications, the majority of commercialized pullulnases type I are of mesophilic and thermophilic origin so far. Hence, the present study underlines cloning, heterologous expression in *Escherichia coli*, characterization, and *in silico* structural modeling of *Metabacillus indicus* open reading frame of cold-adapted pullulanase type I (Pull_Met: 2133 bp & 710 a.a) for the first time ever. The predicted Pull_Met tertiary structure by I-TASSER, was structurally similar to PDB 2E9B pullulanase of *Bacillus subtilis.* Purified to homogeneity Pull_Met showed specific activity (667.6 U/mg), fold purification (31.7), molecular mass (79.1 kDa), monomeric subunit and *Km* (2.63 mg/mL) on pullulan. Pull_Met had optimal pH (6.0) and temperature (40 ^o^C). After 10 h pre-incubation at pH 2.6-6.0, Pull_Met maintained 47.12 ± 0.0–35.28 ± 1.64% of its activity. After 120 min pre-incubation at 30 ^o^C, the retained activity was 51.11 ± 0.29%. At 10 mM Mn^2+^, Na^2+^, Ca^2+^, Mg^2+^, and Cu^2+^ after 30 min preincubation, retained activity was 155.89 ± 8.97, 134.71 ± 1.82, 97.64 ± 7.06, 92.25 ± 4.18, and 71.28 ± 1.10%, respectively. After 30 min pre-incubation with Tween-80, Tween-20, Triton X-100, and commercially laundry detergents at 0.1% (v/v), the retained activity was 141.15 ± 3.50, 145.45 ± 0.20, 118.12 ± 11.00, and 90%, respectively. Maltotriose was the only end product of pullulan hydrolysis. Synergistic action of CA-AM21 (α-amylase) and Pull_Met on starch liberated 16.51 g reducing sugars /g starch after 1 h at 40 ^o^C. Present data (cold-adeptness, detergent stability, and ability to exhibit starch saccharification of Pull_Met) underpins it as a promising pullulanase type I for industrial exploitation.

**Supplementary Information:**

The online version contains supplementary material available at 10.1186/s13568-023-01545-8.

## Introduction

Pullulanases, starch-debranching enzymes (EC 3.2.1.41), have received growing attention by researchers worldwide since they are extensively utilized in the process of starch conversion processes, detergent industry and glucose/maltose saccharification (Samanta 2022). In the context of meeting worldwide enzyme markets demands, novel pullulanases with distinctive catalytic properties under harsh conditions are mandatory and worthy candidates searching for. Theoretically, microbial screening approach is considered the gold standard solution in order to discover novel pullulanase from extremophilic producers. Nevertheless, low pullulanase yield, difficulty in fulfilling the nutritional requirements for extremophiles, inability to mimic the natural and environmental niche conditions for these extremophiles (Lorenz and Eck 2004, van Rossum et al. 2013, Yun and Ryu 2005), and cumbersome multistep downstream purification processes especially concerning pullulanase purification are the utmost obstacles aborting this approach practically and economically (Sarmiento et al. 2015, van Rossum et al. 2013).

Meanwhile, the sequence-based screening approach is a promising alternate toward achieving this goal especially after the continuous revolution in bioinformatics, release of high thorough-put sequencing platforms like next and third generation sequencing, and launching of thousands of projects targeting the full genome sequencing of a plethora of microbes. Hence, the release of millions of raw, curated, and reference putative sequences, deposited in international nucleotide sequences databases (INSCD), does necessitate the obligatory and urgent need to study these putative pullulanase encoding sequences in the lab to unveil the structural-functional relationship encountered in these sequences (Gerlt 2016; Karaiyan et al. 2022; Matrawy et al. 2022a; Pearson 2013).

In the light of the above-mentioned concept, the present study has handled the sequence-based screening approach in order to discover a novel pullulanase as an apparent candidate for industrial exploitation. Intensive navigation in the GenBank to trace novel pullulanases from unstudied bacterial genera did result in selection of *Metabacillus indicus* genome among thousands of pullulanase encoding sequences from a plethora of microbes, to be the source of pullulanase open reading frame in this study.

The genus *Metabacillus* was first introduced taxonomically by Patel and Gupta (2020). The majority of strains assigned to the genus *Metabacillus* were formerly belonging to the *Bacillus* taxa. The current taxonomy of *Bacillus* species considering the genetic diversity, phylogenomic, and comparative genomic approaches, re-classify *Bacillus* species into six novel genera (*Peribacillus* gen. nov., *Cytobacillus* gen. nov., *Mesobacillus* gen.nov., *Neobacillus* gen.nov., *Alkalihalobacillus* gen.nov., and *Metabacillus* gen. nov.). The prefix “meta-“ originates from the Greek adjective *meta*, and refers to “beside”. However, the name of Bacillus does originate from the Latin noun *bacillus*, translating to both ‘a small staff or rod’ and *Bacillus*, the bacterial genus.

Thorough survey in the literature of review indicated that pullulanases from the genus *Metabacillus* had not been investigated yet neither in native form nor in recombinant form. This addressed the indispensable need to unveil the nature of pullulanases from such novel genus namely *Metabacillus.*

The majority of pullulanases are pullulanases type II (Wei et al. 2015). A few studies did investigate pullulanases type I on the gene level such as *Bacillus flavocaldarius* KP 1228 (Abdul-Hadi and Al-Bayyar 2019), *Bacillus thermoleovorans* US105 (Messaoud et al. 2002), *Anaerobranca gottschalkii* (Antranikian et al. 2009), *Caldicellulosiruptor saccharolyticus* (Bielen et al. 2013), *Bacillus* sp. CICIM 263 (Yang et al. 2017), *Thermotoga neapolitana* (Yang et al. 2020) and *Fervidobacterium pennavorans* Ven5 (Yang et al. 2020).

The majority of pullulanases type I under investigation show mesophilic or thermophilic characteristics. There are only a few reports of cold-adapted pullulanases, which address the relatively high catalytic activity at cold temperatures and display optimal activity at moderate temperatures. (Rajaei et al., 2014). As a result of a lower risk of microbial contamination, reduced energy consumption, and the fact that reacting chemicals are frequently unstable at increasing temperatures, cold-adapted enzymes are potential candidates for a variety of biotechnological applications, notably in the food industry (Trincone 2011; Zhang et al. 2020). The aim of the present work is to clone and overexpress enzyme-encoding gene (*pullulanase type I*) of bacterial origin in *E. coli*. The aim is extended to purify, biochemically characterize, and *in silico* analyze the recombinantly expressed enzyme.

## Materials and methods

### Bacterial strains, cultivation conditions, substrates, and reagents

*Escherichia coli* BL21 (DE3) Rosetta (Promega Co., USA) was utilized as the cloning and expression host in this study. Lauria-Bertani (LB) broth was used for the activation and growing purposes of *E. coli* (BL21) DE3 Rosetta strain with an agitation speed of 180 rpm, at 37 °C for overnight. Pullulan (ChemCruz Co., Netherlands), starch, α-amylose, dextrin, and beechwood xylan (Loba Chem, India) were included in this study. Isopropyl-β- D-1-thiogalactopyranoside (IPTG) (MedChemExpress Co., NJ, USA) and dinitrosalysalic acid (Loba Chemie), protein ladder (Bioline, USA) were included in this study. *Glutamicibacter soli* strain AM6 (Obtained as a gift from Dr. Amira Matrawy, Institute of Graduate Studies and Research, Alexandria University, Egypt) was used as the α-amylase (CA-AM21) hyper-producer strain. Lauria Bertani was used as the growth medium unless otherwise stated The α-amylase CA-AM21 production medium consisted of 0.1 KH_2_PO_4_, 0.2 Na_2_HPO_4_, 0.1 NaCl, 0.005 MgSO_4_ 7H_2_O, 0.005 CaCl_2_, 0.2 tryptone, and 1.0 soluble starch) in modified distilled water-based minimal medium (in (w/v) %). The cultivation conditions of *G. soli* were 20 ^o^C and 180 rpm (Brunswick Incubator Shaker, USA).

### Synthesis of recombinant plasmid pET‑28a ( +)/ Pull_Met

The open reading frame (ORF) encoding the *pullulanase* type I gene from *Metabacillus indicus* LMG 22,858, spanning c1647736-1649868 in the whole genome with the accession number JGVU02000002.1, was retrieved from GenBank database. The protein ID reference sequence for the *pullulanase* type I gene locus was KEZ51383.1. The retrieved nucleotide sequence of the *pullulanase* type I gene (2133 bp) was chemically synthesized by GenScript Biotech®. Co., USA (U495WHA200-2) and cloned on the expression vector pET-28a (+). The newly synthesized construct was designated pET-28a (+)/Pull_Met.

### Transformation of pET-28a (+)/Pull_Met into ***E. coli*** BL21 (DE3) Rosetta

The recombinant construct pET-28a (+)/Pull_Met was transformed into chemically competent *E. coli* BL21 (DE3) Rosetta cells as previously reported (Sambrook et al. 1989).

### Recombinant Pull_Met expression in ***E. coli*** BL21 (DE3) Rosetta cells

The transformants *E. coli* BL21 (DE3) Rosetta cells carrying the construct pET-28a (+)/Pull_Met were cultured in a 1 L Erlenmeyer flask containing 200 mL of LB broth supplemented with kanamycin at a final concentration of 34 µg/mL Then, the culture was incubated at 37 °C with an agitation speed of 180 rpm until reaching an optical density of 0.6–0.8 at 600 nm. After that, 1.0 mM isopropyl -D-1-thiogalactopyranoside (IPTG) was added to the culture, and the culture was incubated for a further 18 h at room temperature and 180 rpm. By the end of the induction period, the induced cells were harvested by centrifugation at 6,000×g for 20 min at 4 °C and re-suspended in 4 mL of disruption buffer (50 mM Tris/HCl, pH 7.6; 50 mg/mL lysozyme). A previously described technique (Abady et al. 2022) was applied to break down the induced cells. Concisely, the mixture was incubated for 30 min at 37 °C with gentle shaking. Cell disruption was accomplished via sonication at 14,000 Hz (Fisher Brand TM Sound Enclosure, Thermo Fisher Scientific Co., USA) for 8 cycles of 30 s each, with a two-min pause on ice between the successive cycles. Cell debris was removed by centrifugation at 8,400×g for 15 min at 4 °C. In new Eppendorf tubes, the soluble supernatant of the cell lysate was transferred and then preserved at − 20 °C until further analyses.

### Purification of recombinant expressed Pull_Met

The purification of the recombinantly expressed Pull_Met was carried out using a previously reported procedure with minor modifications (Embaby and Mahmoud 2022; Mahmoud et al. 2021). In brief, the resultant soluble portion of cell lysate containing 100 mg of crude protein was loaded onto a 2 mL Ni^2+^-NTA affinity matrix. Unbound proteins were stripped away from the column by washing it with equilibration buffer (50 mM phosphate buffer, pH 7.5, containing 10 mM imidazole) with five times the bed volume until the absorbance at 280 nm reached zero. After that, washing the column with elution buffer (50 mM phosphate buffer, pH 7.5, containing 500 mM imidazole) eluted the bound 6-His-tagged recombinant Pull_Met protein. The recombinant Pull_Met was eluted in five fractions, each 1 mL. Recombinant Pull_Met activity was assessed using pullulan as the substrate.

### Protein content determination

The Bradford method (Bradford 1976) was used to determine the protein content of the crude soluble cell lysate and the purified fraction. Bovine serum albumin was used to establish a standard curve.

### SDS-PAGE and native -PAGE

The crude cell lysate and purified Pull_Met were subjected to 12% sodium dodecyl sulfate polyacrylamide gel electrophoresis (SDS-PAGE) using the Laemmli method (Laemmli 1970). The molecular weight of recombinant Pull_Met was anticipated using a protein ladder.

The purified Pull_Met was subjected to 10% native-PAGE according to a procedure previously reported (Sonani et al. 2014). The molecular weight of recombinant Pull_Met was anticipated using a protein ladder. In native-PAGE, the sample application buffer lacked SDS and reducing agent. Additionally, the protein sample was not subjected to boiling step prior it’s loading onto the gel. Both SDS-PAGE and native-PAGE were stained with Coomassiae –brilliant blue R-250. Then the gel was stained using de-staining solution (Abady et al. 2022).

### Recombinant Pull_Met activity

As previously reported (Silong et al. 1997), the pull_Met activity was measured colorimetrically by estimating the quantity of liberated reducing sugars using dinitrosalicyclic acid (DNS) reagent (Miller 1959). using pullulan as the substrate. A standard curve of D-glucose was established (Miller 1959). The reaction mixture contained 50 µL pullulan solution (2% (w/v) pullulan in 50 mM sodium phosphate buffer, pH 6.0), and 50 µL crude or purified recombinant Pull_Met. The reaction mixture was incubated at 40 ^o^C for 5 min with agitation at 180 rpm. After that, 1 mL of DNS reagent was added. The tubes were boiled for 5 min. The developed color was measured spectrophotometrically at 540 nm against blank. All enzyme assays were conducted at triplicates. One unit of pullulanase activity was defined as the amount of enzyme that liberates one µmole of D-glucose from pullulan per min.

### Kinetic parameters and substrate specificity

The activity of purified recombinant Pull_Met was assessed on pullulan, starch, α-amylose, dextrin, and beechwood xylan as the substrates. The enzyme assays in the presence of each substrate was measured as mentioned above in case of pullulan with replacement of pullulan with each substrate. One unit of Pull_Met activity on starch, dextrin, and α-amylose was defined as the amount of enzyme that liberated one µmole of D-glucose from each substrate per min. However, one unit of Pull_Met activity on beechwood xylan was defined as the amount of enzyme that liberated one µmole of D-xylose per min.

The Vmax and Km of Pull_Met were determined on pullulan substrate using the Lineweaver–Burk plot, generated by Hyper32 Software.

### Thin layer chromatography (TLC)

The end products of pullulan, dextrin, and starch hydrolysis were tested by thin layer chromatography (TLC). Briefly silica gel plates [0.2 mm silica gel-coated aluminum plates (Kiesel gel 60 F254; Merck, NJ)] using a solvent mixture of *n-*propanol: ammonia: d. H_2_O with a volume ratio 7:1:2 (v:v:v) (Embaby et al. 2018). The end products of pullulan, starch, and dextrin hydrolysis (10 µL) each were loaded in the silica gel plates. Additionally, the substrates pullulan, dextrin, and starch were loaded in the silica gel plates. The standard sugars, glucose and maltose, were loaded in the silica gel plates in parallel. The spots were visualized by spraying the plates with a solution of diphenylamine and aniline (1gram diphenylamine and 1 mL of aniline in 100 mL acetone, then this mixture is further mixed with 85% orthophosphoric acid prior to use (10:1 v/v). Then, the plates were charring until the spots became visible.

### Biochemical characterization of recombinant Pull_Met

Pullulan was used as the substrate for all Pull_Met biochemical characterization assays. All reactions were carried out in triplicate. The values were provided as the mean of three replicates with standard error (SE).

#### pH and temperature optima

The optimal pH was established across a wide pH range of 2.6–10.6: 50 mM acetate buffer for pH (s) 3.0, 4.0, 5.6, 50 mM citrate buffer for pH(s) 5.6, 6.0 & 50 mM phosphate buffer for pH(s) 6.0, 7.0, 8.0 & 50 mM Tris-HCl buffer for pH(s) 7.0, 8.0, 8.5, 9.0 & 50 mM Glycine- buffer for pH(s) 9.0, 10.0, 10.6, and 50 mM carbonate buffer for pH(s) 10, 10.6. All enzyme assays using different buffers were conducted at 40 ^o^C.

The optimal temperature of Pull_Met was determined at several temperatures ranging from 5 to 60 °C. The control reaction was the enzyme activity evaluated without any pretreatment.

#### Temperature and pH stability

The thermal stability of Pull_Met was investigated by measuring residual activity after incubating the enzyme at different temperatures (15–45 °C) in 50 mM sodium phosphate buffer, pH 6.0, at three-time intervals; 30, 60, and 120 min. After that, the reaction tubes were placed on ice for 5 min prior performing enzymatic assays at the optimal temperature. The residual activity was determined at each tested pH.

The influence of pH on Pull_Met stability was determined by incubating the enzyme at 4 °C overnight in the aforementioned buffers ranging from 2.6 to 10.6. Following the completion of the incubation period, enzyme assays were performed. The residual activity was determined at each tested temperature.

#### Effect of metal ions, detergents, organic solvents and inhibitors

The influence of different metal ions on Pull_Met stability was estimated by pre-incubating the enzyme in the presence of different metal ions; Mn^2+^, Mg^2+^, Na^+^, Ca^2+^, Cu^2+^, Zn^2+^, K^+^, Fe^3+^, Hg^2+^, and Ni^2+^ for 30 min at two tested concentrations 5.0 and 10 mM for each metal ion.

The stability of recombinant Pull_Met in the presence of detergents was evaluated by pre-incubating the enzyme with Tween 20, Tween 80, Triton X-100, CTAB and SDS at three tested concentrations, 0.1, 0.25, and 0.5 (v/v %) for 30 min at 4 °C in 50 mM Sodium phosphate buffer, pH 6.0.

The effect of some commercially available laundry detergents (Ariel^®^, Oxi^®^, Persil^®^, Art^®^, and Tide^®^) on Pull_Met stability was determined according to a previously reported procedure (Matrawy et al. 2022).

The influence of solvents on recombinant Pull_Met stability was estimated after 30 min pre-incubation of the enzyme in 10 and 20% (v/v) solutions of ethanol, methanol, *n*-butanol, isopropanol, and acetone at 4 ^o^C.

The impact of β-mercaptoethanol and ethylene diamine tetra acetic acid (EDTA) on Pull_Met stability was investigated after 30 min pre-incubating the enzyme at two tested concentrations 5 and 10 mM for each.

In all investigations, after 30 min pre-inucbation with the effector, the Pull_Met activity was conducted at the optimal temperature and pH. Moreover, the residual activity of Pull_Met was estimated in the presence of each effector in relation to the control reaction (without effector).

### Applications of pullulanase

For starch saccharification, the co-operative action between the starch-degrading enzyme (CA-AM21) (Matrawy et al. 2022) and Pull_Met was studied. The partially purified CA-AM21 α-amylase was prepared and assayed as previously mentioned (Matrawy et al. 2022). The synergistic interaction of Pull_Met with the partially purified α-amylase (CA-AM21) (35.2 µg protein having 21.28 U/mg) on the hydrolysis of raw ex potato starch was tested at 40 °C for 1 h using varying Pull_Met dosages (0, 1.9, 3.8, 5.7, 7.6, 9.5, 11.4, and 13.3 µg protein). After 1 h, the liberated reducing sugars were determined using DNS reagent (Miller 1959). The reducing sugars liberated from the conversion of starch by the synergistic effect of CA-AM21 and Pull_Met were expressed in terms of gram reducing sugars/gram starch substrate. The experiment was conducted in triplicates.

#### *In silico* Pull_Met sequence analyses

The Signal IP 6.0 server (http://www.cbs.dtu.dk/services/SignalP/) (Petersen et al. 2011) was used to predict the presence of N-terminal signal peptide in the Pull_Met amino acid sequence. The translated protein amino acid sequence of Pull_Met was obtained via Expasy, the Swiss Bioinformatics Resource Portal, located on the server (https://web.expasy.org/translate/). The nucleotide sequence of Pull_Met and its translated protein amino acid sequence were searched against the non-redundant nucleotide collection database and UniProtKB/SwissProt (Swissprot) using the BLASTN and BLASTP online programs (https://blast.ncbi.nlm.nih.gov/Blast.cgi?PAGE=Proteins), respectively.

The secondary structure of the translated Pull_Met protein was predicted using the SAS server (https://www.ebi.ac.uk/thornton-srv/databases/sas/).

The multiple sequence alignment of the Pull_Met amino acid sequence and those of pullulanases from other species and the phylogenetic tree depicting the evolutionary relationships of aligned sequences were done by MEGA 7.0. The three-dimensional (3D) structure of the Pull_Met protein was predicted using I-TASSER located at the server https://zhanggroup.org/I-TASSER/ (Zheng et al. 2021). The PyMOL (Schrödinger, LLC, Portland, OR) was used to display the molecular image of the predicted 3D structure of Pull_Met included in the PDB file, which was retrieved from the output of the I-TASSER online program. The theoretical pI, molecular mass, and amino acid composition of Pull_Met were inferred from the web-tool ProtParam located at the server (https://web.expasy.org/protparam/).

The presence of any transmembrane helices in the Pull_Met protein was predicted by the three online programs: TMHMM 2.0 (https://services.healthtech.dtu.dk/service.php? TMHMM-2.0), SOSUI (https://harrier.nagahama-i-bio.ac.jp/sosui/mobile ) (Mitaku et al. 2002), and PHOBIUS (https://www.ebi.ac.uk/Tools/pfa/phobius).

## Results

### Cloning, expression, and purification of Pull_Met

The full-length of *pullulanase* type I ORF (2133 bp) from *Metabacillus indicus* LMG 22,858, with the protein ID: KEZ51383.1, was retrieved from GenBank and chemically synthesized by GenScript Co. The chemically synthesized *pullulanase* gene was successfully cloned in pET-28a (+) expression vector. The cloned *pullulanase* gene was successfully overexpressed in the *E. coli* BL 21(DE3) Rosetta cells. The recombinantly expressed protein of 710 amino acid residues was designated Pull_Met. The nucleotide sequence of the Pull_Met encoding-gene was deposited in GenBank under the accession number OP585545.1. Pull_Met was expressed in a soluble active form in 1 mM IPTG induced recombinant *E. coli* BL21(DE3) Rosetta cells harboring the construct pET-28a ( +) /Pull_Met at 37 ^o^C after 18 h of induction at 180 rpm.

**Fig. 1:**
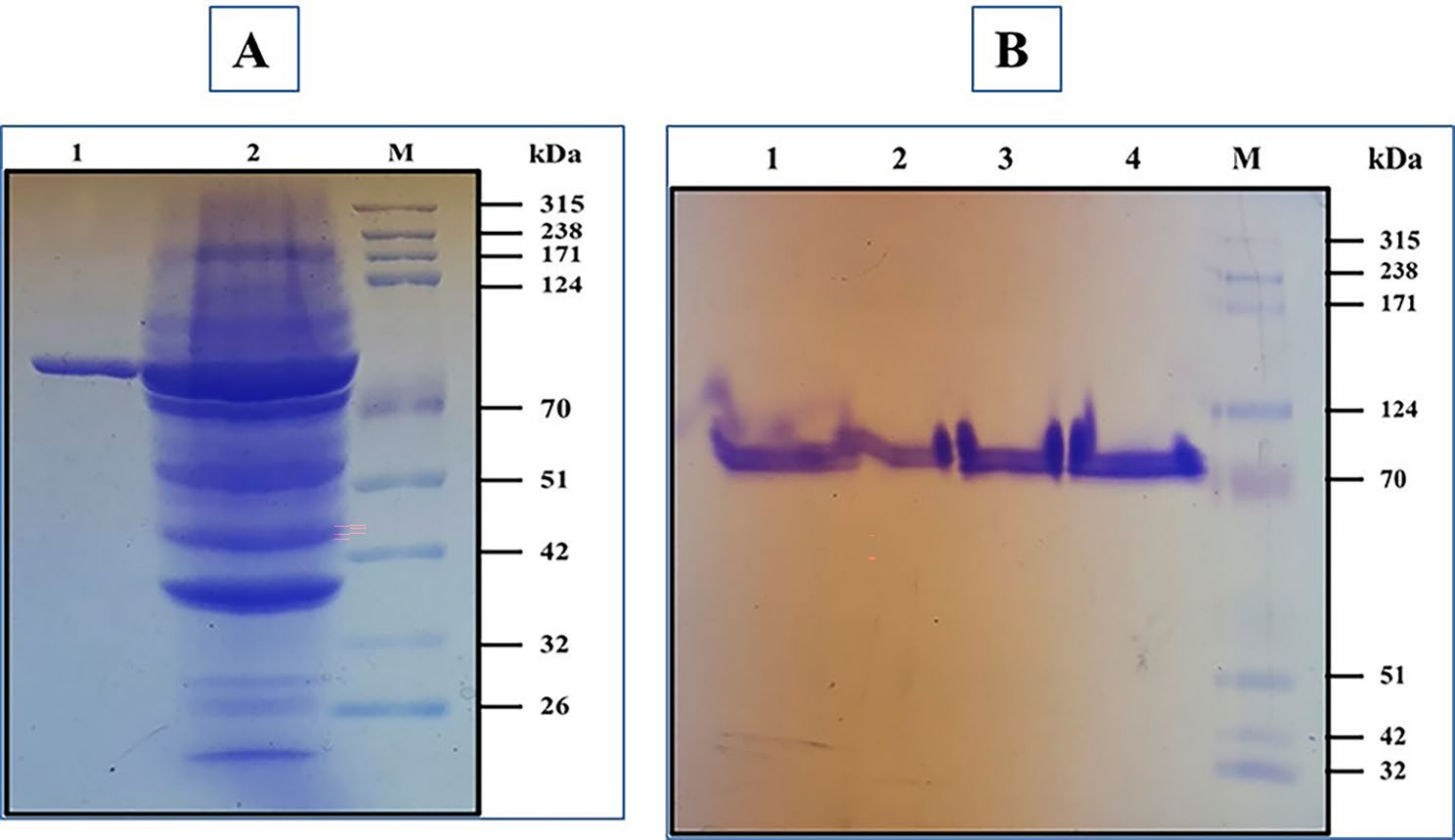
** A)** 12% SDS-PAGE showing purification steps of Pull_Met using Nickel -affinity agarose chromatography. Lane M: Protein ladder. Lane 1: purified Pull_Met after Nickel -affinity agarose chromatography column. Lane 2: crude cell lysate of induced recombinant *E. coli* BL21 (DE3) Rosetta cells. **B)** 10% native-PAGE showing the multimerization status of Pull_Met. Lane M: Protein ladder. Lanes (1–4): Pull_Met purified to homogeneity using nickel affinity chromatography column.

The recombinant expressed Pull_Met was successfully purified to homogeneity using Nickel agarose affinity column chromatography (Table S1). The Pull_Met was purified with specific activity, fold purification, and recovery of 667.6 U/mg, 31.7, and 98.3%, respectively. SDS-PAGE revealed that the recombinant enzyme had a molecular mass of approximately 79.1 kDa as a single band (Fig. 1A). Both SDS-PAGE and native –PAGE (Fig. 1B) revealed that the Pull_Met was monomeric with a molecular mass of 79.1 kDa.

### Biochemical characterization of recombinant Pull_Met

**Fig. 2:**
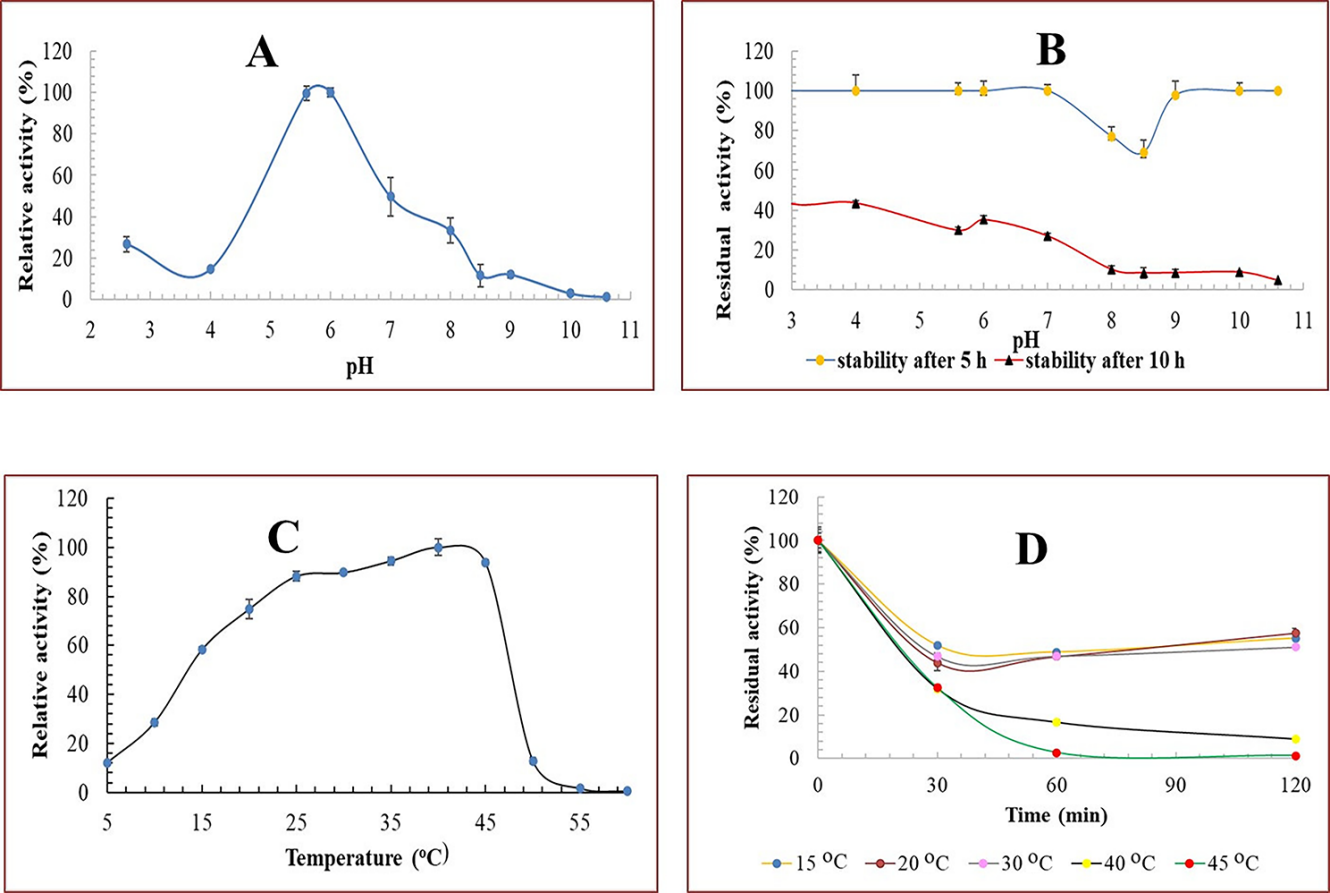
pH-temperature profile of purified to homogeneity Pull_Met. **A)** Pull_Met activity profile over pH range 2.6–10.6. **B**) pH stability profile of Pull_Met over pH range 2.6–10.6. **(C)** Pull_Met activity profile over temperature range 5–60 °C. **(D)** Temperature stability profile of Pull_Met over temperature range 15–45 °C. Results are the mean of experimental readings performed in triplicate ± SE.

Data revealed that Pull_Met exhibited appreciable activity in different buffer system from pH 2.6-9 as depicted in Fig. 2A. The ultimate activity of Pull_Met was realized at pH 6.0 using phosphate buffer. A significant decline in Pull_Met activity was noticeable at extremist pH(s): acidic side (2.6- 5.0) and alkaline side (8.0-10.6). The lowest relative activity of Pull_Met (26.86 ± 3.5, 14.63 ± 0.03, 11.71 ± 5.41, 12.09 ± 1.58, 3 ± 1.39, and 1.14 ± 0.31%) exhibited at pH(s) 2.6, 4.0, 8.5, 9.0, 10.0, and 10.6, respectively.

Pull_Met activity was almost fully stable (100%) at pH(s) 2.6, 4.0, 5.6, 6.0, 7.0, 9.0, 10.0, and 10.6 after 5 h of pre-incubation. Whilst, moderate stability (77.08–69.18%) at pH(s) 8.0, and 8.5 was retained after 5 h (Fig. 2B). Conversely, after 10 h of pre-incubation at pH(s) 2.6-6.0, Pull_Met maintained 47.12 ± 00–35.28 ± 1.64% of its activity. A dramatic significant reduction in Pull_Met activity was retained after 10 h at pH(s) 7-10.6 (Fig. 2B).

Data showed that the optimal temperature for Pull_Met activity was at 40 ^o^C (Fig. 2C). Pull_Met showed its highest activity (88.24 ± 2.1–100%) at a range of temperatures from 25 to 40 ^o^C. Conversely, a dramatic significant decline in Pull_Met activity (12.15 ± 0.4, 28.62 ± 1.5, 12.88 ± 1.32, 1.57 ± 0.15, and 0.43%±0.1) was observed at extremist temperatures 5, 10, 50, 55, and 60 ^o^C, respectively.

Around 50% of Pull_Met activity was retained at 15, 20, and 30 ^o^C (Fig. 2D) along 2 h. However, a significant dramatic decline in Pull_Met activity (8.86 ± 20.9 and 1.29%±0.02) was observed at 40 and 45 ^o^C, respectively after 2 h.

**Fig. 3:**
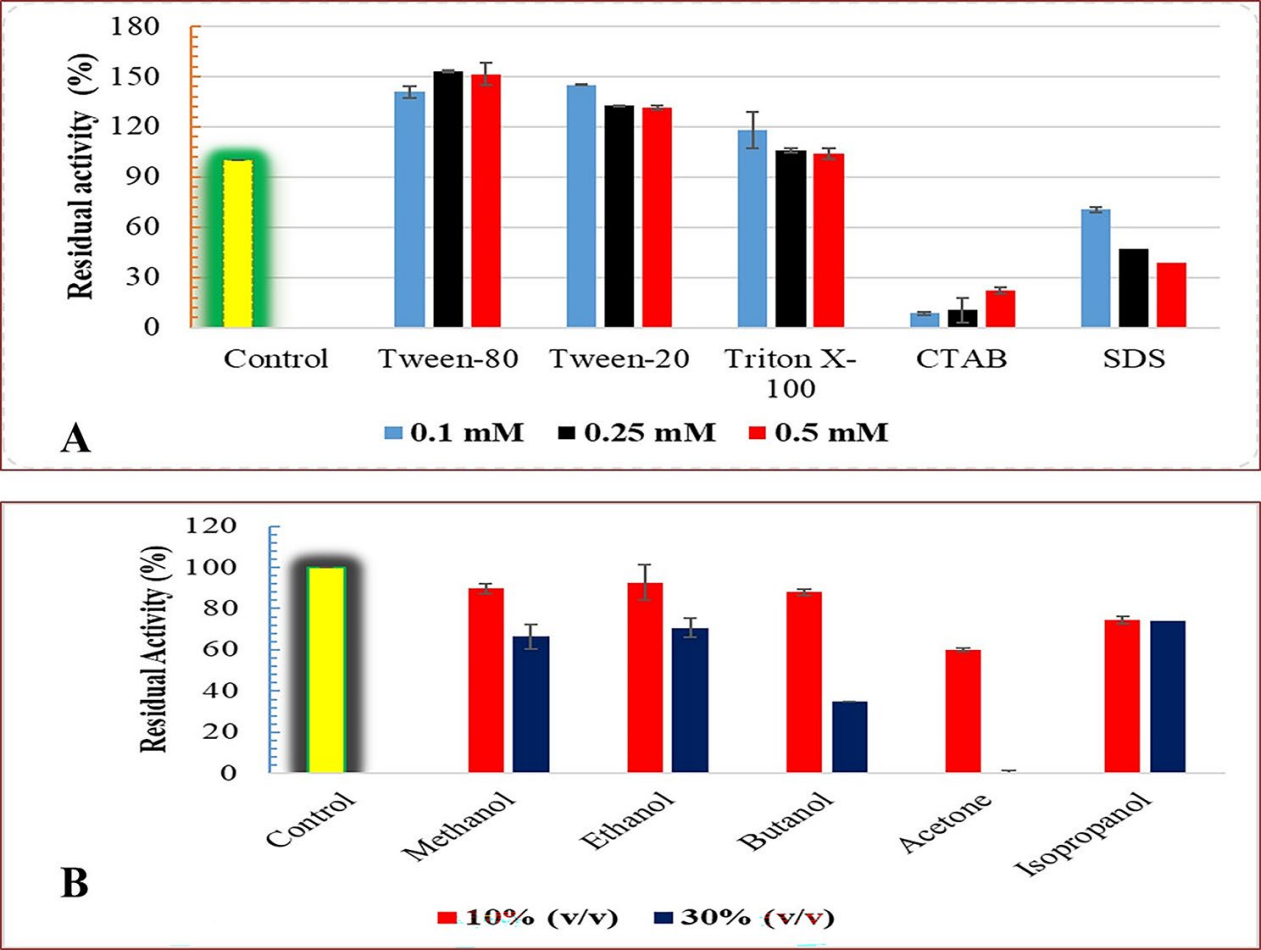
** A)** Stability profile of Pull_Met in presence of some detergents at three tested concentrations: 0.1, 0.25, and 0.5 mM. Control: without any pretreatment with detergents. Values are the average of three readings ± SE. **B)** Pull_Met stability in presence of solvents. Values are the average of three readings ± SE.

The stability of Pull_Met in the presence of non-ionic detergents (Tween-80, Twen-20, and Triton X-100) and ionic detergents (CTAB and SDS) at three tested concentrations 0.1, 0.25, and 0.5 mM were investigated after 30 min pre-incubation (Fig. 3A). A significant enhancement in the Pull_Met activity (up to 151%) was noted in the presence of Tween-80 at 0.25 and 0.5 mM. Similarly, Tween-20 exhibited the same effect on Pull_Met activity with a significant enhancement in the activity (up to 131%) at 0.25 and 0.5 mM. Conversely, a significant dramatic decline in Pull _Met activity up to 22.36% was observed after 30 min pre-incubation with CTAB. Regarding SDS, the retained Pull_Met activity was 47.11 ± 7.09 and 38.9% ± 2.02, respectively at 0.25 and 0.5 mM.

The stability of Pull_Met in the presence of some commercially laundry detergents after 30 min pre-incubation was tested (Table 1). Data revealed that Pull_Met retained the majority of its activity (around 98%) with Oxi ^TM^ and Tide ™. However, around 89% of activity was retained with Persil ™, Ariel ™, and Art ™.

**Table 1:** Purification table of Pull_Met using nickel-agarose affinity matrix

Purification step	Total Units	Total mg protein	Specific activity (U/mg)	Fold *	Recovery (%)
Crude cell lysate	915.00	43.50	21.03	1.0	100
After nickel affinity agarose chromatography column	900.0	1.33	667.60	31.7	98.3

*Fold = specific activity at any step/specific activity at initial step

The stability of Pull_Met after 30 min of pre-incubation with some solvents at 10 and 30% (v/v) was studied (Fig. 3B). Almost 90% of Pull_Met activity was retained after 30 min pre-incubation of the enzyme with 10% (v/v) of methanol, ethanol, and butanol. While, almost 70% of Pull_Met activity was retained after 30 min pre-incubation of the enzyme with 30% (v/v) of methanol, ethanol, and isopropanol. Full loss of Pull_Met activity occurred after 30 min of pre-incubation in 30% (v/v) acetone.

The stability of Pull_Met in the presence of some metal ions after 30 min pre-incubation was studied (Table 2a). Data verified that Mn^ 2+^, Na^+^, and K^+^ exhibited significant stimulatory effect on Pull_Met activity at both tested concentrations (5 and 10 mM). The attained Pull_Met activity was 155.89 ± 8.97, 134.71 ± 1.82, and 145.15 ± 5.04% after 30 min pre-incubation with 10 mM Mn^ 2+^, Na^+^, and K^+^, respectively. Whilst, 30 min pre-incubation of Pull_Met with Mg^ 2+^ did not impose neither significant enhancement nor decline in activity. However, 97.64 ± 7.06% of Pull_Met activity was retained after 30 min pre-incubation with 10 mM Ca^+ 2^. The retained Pull_Met activity in the presence of Zn^ 2+^, Fe^ 2+^, and Cu^ 2+^ was 74.09 ± 2.84, 79 ± 9.03, and 82.41 ± 5.9% at 5 mM each, respectively. A significant dramatic decline in Pull_Met activity was noticed in the presence of Hg^ 2+^ and Ni^ 2+^ with retained activity of 20.17 ± 1.25 and 0.00% at 5 mM each, respectively.

**Table 2:** **a**: Stability of Pull_Met in presence of some metal ions, β-mercaptoethanol, and EDTA

	Residual activity (%)	
Control^a^	100.00	
Metal ion	(5 mM)	(10 mM)
MnSO_4_	150.53 ± 4.25	155.89 ± 8.97
NaCl	184.47 ± 5.61	134.71 ± 1.82
CaCl_2_	93.03 ± 2.20	97.64 ± 7.06
CuSO_4_	82.41 ± 5.90	71.28 ± 1.10
ZnCl_2_	74.09 ± 2.84	68.24 ± 0.12
KCl	140.05 ± 0.70	145.15 ± 5.04
FeSO_4_	79.00 ± 9.03	51.23 ± 0.54
Mg SO_4_	112.92 ± 9.40	92.25 ± 4.18
HgCl_2_	20.17 ± 1.25	28.04 ± 1.27
NiSO_4_	0.00	0.00
β-mercaptoethanol	173.79 ± 4.20	202.91 ± 5.61
EDTA	18.60 ± 0.61	23.78 ± 0.83

Readings are the average of three readings ± SE.

**Table 2:** **b**: Stability of Pull_Met in presence of commercially available laundry detergents

Commercially available laundry detergents	Residual activity (%)
Control^a^	100.00
Oxi ^TM^	98.72 ± 1.00
Tide ^TM^	98.37 ± 1.00
Persil ^TM^	90.75 ± 0.40
Ariel ^TM^	89.80 ± 0.25
Art ^TM^	88.14 ± 0.31

Readings are the average of three readings ± SE.

The stability of Pull_Met after 30 min pre-incubation of β-mercaptoethanol and EDTA at two concentrations 5 and 10 mM was tested (Table 2b). Data conferred that β-mercaptoethanol at 10 mM imposed a significant increase in Pull_Met activity reaching a value of 202.91 ± 5.61%. On the other hand, Pull Met activity was dramatically reduced by EDTA at both of the tested concentrations, reaching values of 18.6 ± 0.61 and 23.78 ± 0.83%, respectively.

The specificity of Pull_Met towards different substrates was tested as demonstrated in Table 3. Pull_Met exhibited its utmost activity on pullulan as the substrate (28.92 U/mg). The pull_Met substrate specificity could be outlined in the following order: pullulan > dextrin > starch > beechwood xylan > α-amylose.

**Table 3:** Substrate specificity of Pull_Met

Substrate	Relative specific activity (%)
Pullulan	100 ± 0.00
Dextrin	42.97 ± 1.30
Starch	17.47 ± 0.03
Beechwood xylan	4.52 ± 0.68
α-amylose	1.145 ± 0.22

Readings are the average of three readings ± SE.

**Fig. 4:**
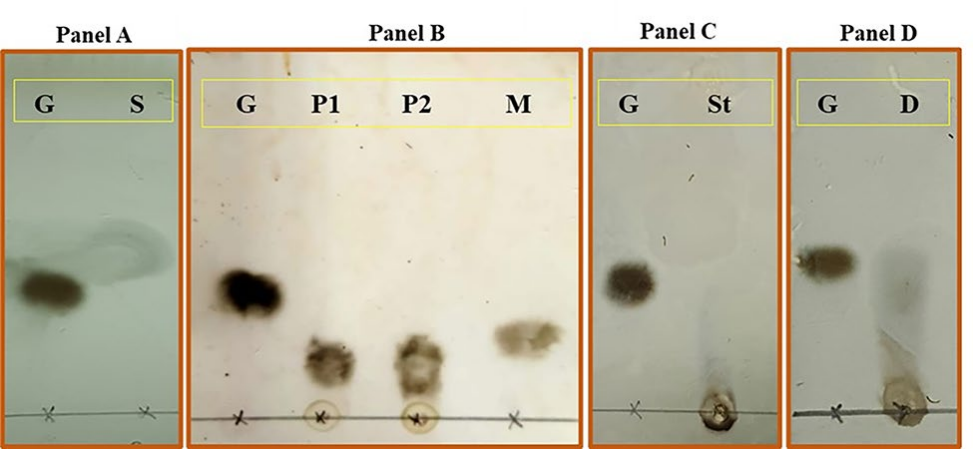
TLC showing pullulan end products hydrolysis by Pull_Met. Panel **A**: G: glucose standard. S: undigested pullulan substrate. Panel **B**: G: glucose standard, M: maltose standard, P1: pullulan end products of hydrolysis (5 µL) and P2 pullulan end products of hydrolysis (10 µL): by Pull_Met. Panel **C**: G: glucose standard. St: standard end products of starch hydrolysis. Panel **D**: G: glucose standard, **D**: Dextrin end products of hydrolysis.

The kinetics parameters Km and Vmax were determined for Pull_Met on pullulan. The kinetic parameters were 2.369 mg/mL and 3.313 µmole glucose. min^− 1^, for Km and Vmax, respectively. The end products of pullulan, dextrin, and starch by the action of Pull_Met was analyzed on thin layer chromatography (TLC). Only maltotriose as the end product of pullulan hydrolysis by Pull_Met was detected on TLC plate (Fig. 4, panel B). No detectable end products of dextrin (Fig. 4, panel D) and starch (Fig. 4, panel C) hydrolysis could be observed on TLC plate.

## Pull_Met potential in starch saccharification

**Fig. 5:**
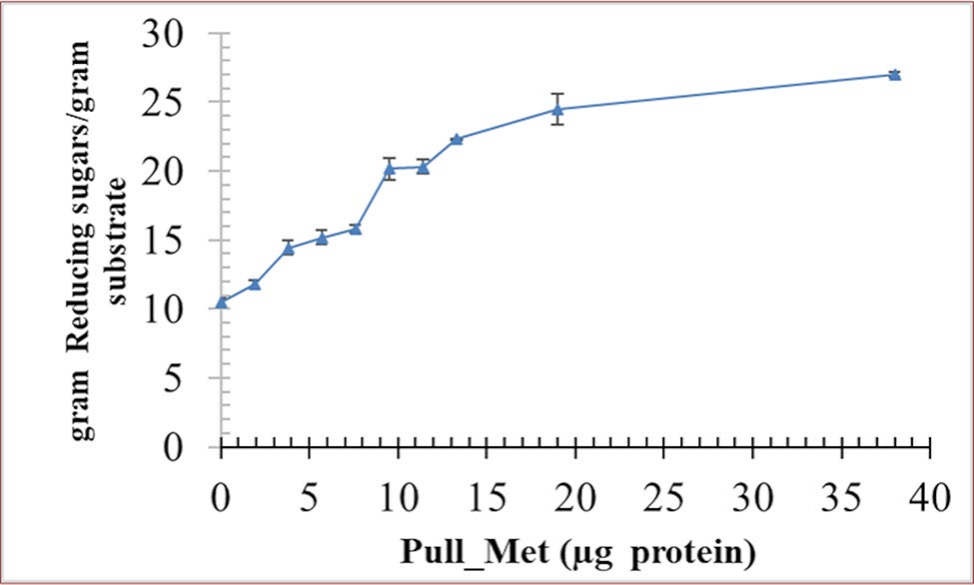
Synergistic action of gradual concentrations of Pull_Met with 21.28 U/mg CA-AM21 on raw ex potato starch saccharification. Values are the average of three readings ± SE.

Pull_Met exhibited potential in raw ex potato starch saccharification in synergistic co-operative action with CA-AM21 as concluded from the liberated reducing sugars in terms of glucose from the raw starch (Fig. 5). The CA-AM21 (21.28 U/mg) action in solitary on starch resulted in the liberation of 10.49 ± 0.25 g reducing sugars/gram starch substrate after 1 h incubation at 40 ^o^C. Whilst, the combined (synergistic) action of CA-AM21 and different concentrations of Pull_Met (0, 1.9, 3.8, 5.7, 7.6, 9.5, 11.4, and 13.3 µg) resulted in a significant gradual increase in the liberated reducing sugars (Fig. 5). The ultimate net reducing sugars resulted from synergistic action of CA-AM21 (21.28 U/mg) and Pull_Met (13.3 µg) was 16.51 g/gram starch substrate after 1 h incubation at 40 ^o^C.

### Structural modeling of Pull_Met

A BLASTp sequence similarity search against the non-redundant database revealed that the translated Pull_Met amino acid sequence had high similarity identities with pullulanase (WP_029278928.1) of *Metabacillus indicus* and other pullulanases from other species belonging to the genus *Bacillus* (Table S1).

This genetic relatedness between Pull_Met amino acid sequences and other homologous pullulanase sequences was unraveled by the phylogenetic tree constructed by MEGA 7.0 using Neighbor –joining method (Figure S1).

**Fig. 6:**
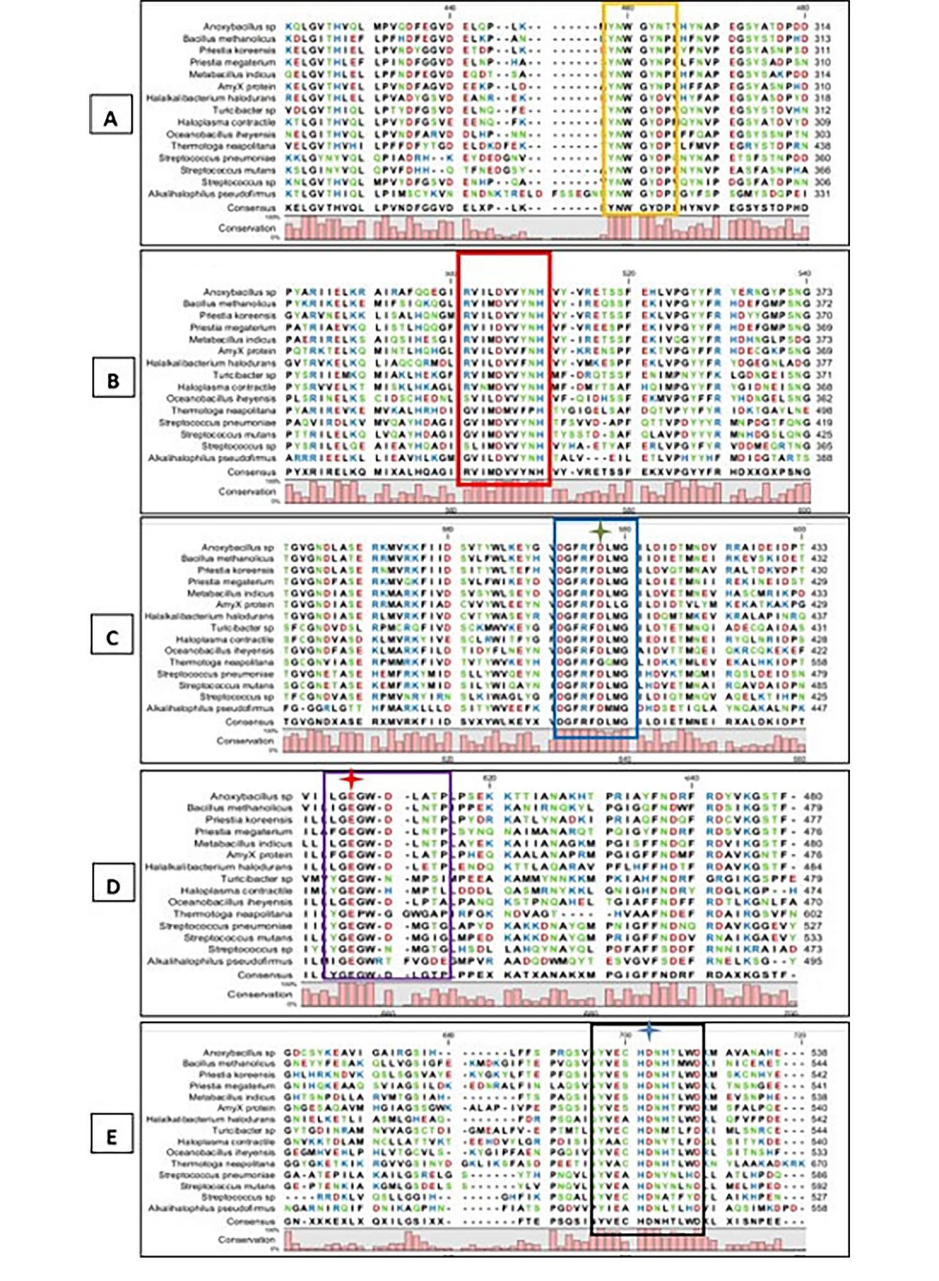
Multiple sequence alignment between Pull_Met and other pullulanases type I from other species. **A**: the amino acid sequence of pullulanase type I signature motif highlighted by rectangle. **B**: the amino acid sequence of conserved region I in GH13 family highlighted by rectangle. **C**: the amino acid sequence of conserved region II in GH13 family highlighted by rectangle. **D**: the amino acid sequence of conserved region III in GH13 family highlighted by rectangle. **E**: the amino acid sequence of conserved region IV in GH13 family highlighted by rectangle. The accession number of pullulanases were AEW23439.1 (*Anoxybacillus* sp.), WP_004439017.1 (*B. methanolicus*), WP_251605681.1 (*Priestia koreensis*), UJT32179.1 (*Priestia megaterium*), UZX50359.1 (*M. indicus*), 2E8Y (AMyx protein of *Bacillus cereus*), WP_068759737 (*Turicibacter* sp.), WP_010899405.1 (*Halakalibacterium halodurans*), WP_011064811.1 (*Oceanobacillus iheyensis*), WP_008826932.1 (*Haloplasma contractile*), ACN58254.1 (*Thermatoga neapolitana*), WP_002277520.1 (*Streptococcus mutans*), WP_000283071.1 (*Streptococcus pneumoniae*), WP_067193616.1 (*Strreptocccus* sp.), and AVI10282.1 (*Alkalihalophilus pseudofirmus*). The catalytic triad residues: Asp, Glu, and Asp are indicated by asterisks

Data of MSA revealed the presence of five signature motifs and domains: conserved motif of pullulanases type I and four regions I, II, III, and IV of GH13 family (Fig. 6). The amino acid sequence of the pullulanase type I signature motif was YNWGYNP. While the amino acid sequence of the four conserved regions of GH13 was region I (RVIIDVVYNH), region II (DGFRFDLMG), region III (FGEGWDLNTP), and region IV (YVESHDNTLWD). The catalytic triad residues of Pull_Met were Asp ^410^, Glu^439^, and Glu^523^ as shown in Fig. 6.

The localization of the Pull_Met inside the microbial cell was predicted by analyzing the amino acid sequence through two online programs: TMHMM and Phobius. Analysis of the amino acid sequence of Pull_Met by the two aforementioned programs revealed that Pull_Met was an extracellular protein as depicted in Figure S2 and Figure S3.

The secondary structure of Pull_Met was predicted by SAS online program as portrayed in Figure S4. The built secondary structure of Pull_Met was predicted based on the crystal structure of pullulanase (PDB: 3Wdh: A) from *Anoxybacillus* sp. Lm18-11 as a template with identity percent of 52.5%. As depicted in Figure S4, the predicted secondary of Pull_Met showed 31 helices and 34 β-sheets.

**Fig. 7:**
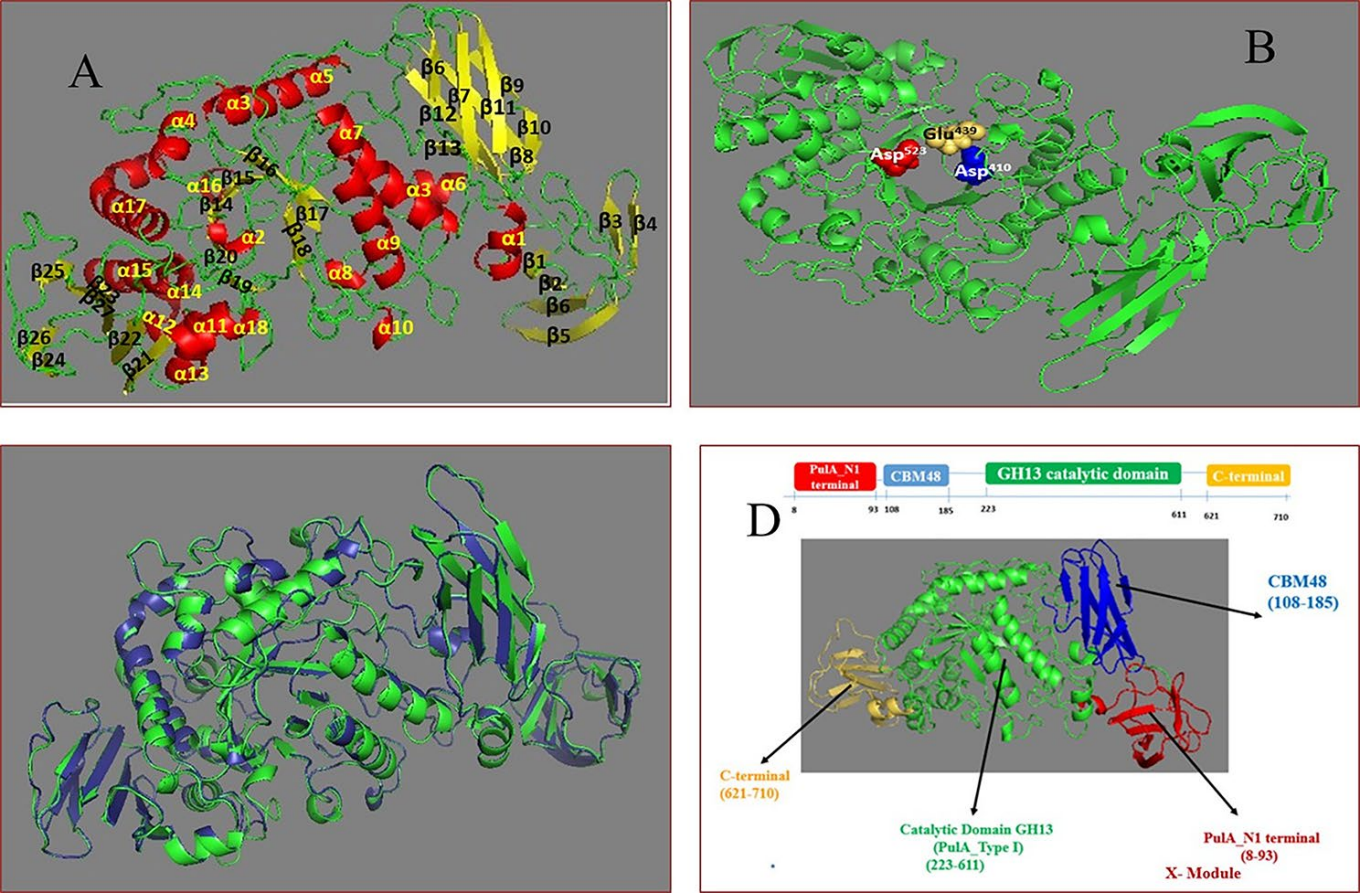
** A)** Predicted 3D structure of Pull_Met, generated by I-TASSER online program and visualized by PyMOL program, in a cartoon view showing α/β fold hydrolase using crystal structure PDB 2E9B of pullulanase type I from *Bacillus subtilis* str. 168. **B)** Cartoon view of predicted 3D structure of Pull_Met showing the catalytic triad residues at Asp ^410^ (in blue spheres), Glu ^439^ (in yellowish orange spheres), and Asp ^523^ (in red spheres). **C)** Superposition, generated by TM-align Structural Alignment program and visualized by PyMOL program, between predicted 3D structure of Pull_Met (blue) and PDB hit 2e9b (green) in a cartoon view. **D)** Schematic representation of Pull_Met showing predicted domain organization as deduced c analysis of amino acid sequence on InterPro online program.

The 3D (tertiary) structure of Pull_Met (Fig. 7A) was predicted by I-TASSER online program using the MODELLER LOMETS S3. The predicted model was built based on the PDB hit: 2e9b (pullulanase type I of *Bacillus subtilis* str.168) exhibited the highest sequence and structural similarity with Pull_Met as deduced from 10 threading programs of LOMETS S3 and TM-align (structural alignment program), respectively.

The 3D predicted structure indicated 18 α- helices and 27 β-sheets in α/β fold like hydrolases. The catalytic triad residues Asp ^410^, Glu ^439^, and Asp ^523^ were localized on the predicted 3D structure (Fig. 7B).

The 3D structure of the PDB hit 2e9b, showing the highest structural similarity with Pull_Met, was superimposed with the 3D structure of Pull_Met as demonstrated in Fig. 7C. The RMSD (Root mean square deviation) between residues of Pull_Met and 2E9b that are structurally aligned by TM-align was 0.39, reflecting the highest structural similarity between the two 3D structures. As a rule of thumb, the highest similarity between the two superposed 3D structures is represented by the smallest value of RMSD. Analysis of Pull_Met amino acid sequence on InterPro online program showed that Pull_Met has four domains: PulA_N1 terminal, carbohydrate binding module 48 (CBM48), glycosidic hydrolase 13 (GH13) catalytic domain, and C-terminal spanning from the following amino acids: 8–93, 108–185, 223–611, and 621–710, respectively (Fig. 7D).

## Discussion

The present study does handle the cloning, heterologous expression, biochemical characterization, and *in silico* structural modeling of pullulanase type I from *Metabacillus indicus* for the first time ever. The molecular structure of Pull_Met was similar to the structure of other pullulanases homologues from other species such as pullulanases type I with the PDB entries: 2WAN, 2WDJ, 2E8Z, and 2YDT from *Bacillus acidopullulyticus*, *Anoxybacillus* sp. LM18-11, *B. subtilis* str.168, and *S. pnumoniae*, respectively. The aforementioned homologues pullulanases and Pull_Met molecular structures did share a uniform architecture with multiple domains: N-domain, CBM (carbohydrate binding module), catalytic domain, and C-domain (Xu et al. 2021). These CBM (e.g., CBM41, CBM48, and CBM68) frequently reported in the architecture of glycosidic hydrolases of family GH13, play a crucial role in the binding of pullulanases to the starch/glycogen and help facilitate the hydrolysis process (Janeček et al. 2017). At most, pullulanases type I CBM (s) like CBM41 and CBM48 are disordered with several modules of unveiled functions termed X modules. For instance, pullulanase type I of the PDB entry 2WAN had CBM 41 and CBM48 disordered with X-modules namely X25 and X45 of unknown functions. However, for Pull_Met and its structurally similar PDB entry 2E9B of *B. subtilis* str. 168 pullulanase type I, the architecture is much simpler as only X25 module of 104 residues preceding the CBM48 (Turkenburg et al. 2009).

The conserved motif of seven amino acids YNWGYNP was reported to predominate in all pullulanases pullulanase type I that only could attack α-1,6 glycosidic bonds of pullulan (Erden-Karaoğlan et al., 2019, Iqrar et al. 2020, Prongjit et al. 2022), this region would appear to be involved in substrate binding or catalytic activity (Bertoldo et al. 1999, Yamashita et al., 1997). Reportedly, this conserved motif does not exist in pullulanases type II that could attack both α-1,4 glycosidic bonds and α-1,6 glycosidic bonds in pullulan.

Likewise other pullulanases type I, belonging to GH13, Pull_Met had four conserved regions namely I, II, III, and IV localized in the catalytic cleft for substrate binding as shown in Fig. 6. The catalytic triad residues Asp^410^, Glu^439^, and Asp^523^ of Pull_Met were located on β-strand, β-strand, and loop in the β/α barrel, respectively. Whilst, the catalytic triad (Asp, Glu, and Asp) of other pullulanases type I (e.g., 2FH8, 2YA1, 2WAN, 6JEQ, 3WDJ, and 2E8Z) were reported to be localized all on β-strands (Xu et al. 2021). Reportedly, in GH13 pullulanases, the *β*-4-Asp, *β*-5-Glu, and *β*-7-Asp represented the catalytic nucleophile, proton donor, transition-state stabilizer, respectively.

The Pull_Met amino acid sequence lacked a signal peptide as deduced from Signal IP 6.0 server. Similarly, the amino acid sequences of pullulanases type I such as 2FH8, 2YA1, 2WAN, 6JEQ, 3WDJ, and 2E8Z did lack signal peptide sequence as revealed from the analysis on Signal IP 6.0 server. Conversely, pullulanase type I of *Fervidobacterium pennavorans* Ven5 showed a signal peptide of 28 amino acid residues (Bertoldo et al. 1999). As a rule of thumb, the signal peptide plays an essential role in protein secretion outside the cell. Nevertheless, the heterologous expression of majority of reported pullulanases type I have not realized high expression levels and effective extracellular secretion despite the presence of signal peptide sequence or secretory proteins as fusion partners (Albertson et al., 1997, Michaelis et al. 1985, Takizawa et al. 1985, Tomiyasu et al., 2001). Conversely, the pullulanase type I from *Klebsiella pnumoniae* 342, *K. variicola* AT-22, and *K. variicola* SHN-1 showed a signal peptide (Chen et al., 2013).

The molecular mass of Pull_Met (79.1 kDa) was localized in the range of reported molecular masses of pullulanases type I (70–140 kDa). For instance, the molecular masses of pullulanases type I from *Anaerobranca gottschalkii* (Bertoldo et al. 2004), F. *pennavorans* Ven 5 (Hii et al. 2012), *Clostridium thermohydrosulfuricum* (Saha et al. 1988),, *Bacillus* sp. S-1 (Lee et al. 1997), *Thermatoga maritima* (Kriegshäuser et al., 2000), and *Bacillus naganoensis* (Zhang et al., 2013) were 96, 77, 136.5, 80, 140,89, and 100 kDa, respectively.

Regarding the multimerization status (quaternary structure) of Pull_Met, the experimental data derived from native –PAGE (monomeric subunit) was in a good accordance with that of predicted data derived from SWISS-MODEL. Moreover, the quaternary structure of Pull_Met was in a good agreement with that of the majority of previously reported pullulanases type I (e.g., *Exiguobacterium acetylicum* (Qiao et al., 2015) and *L amylophillus* GV6 ) (Dakhmouche Djekrif et al. 2021), with the exception of pullulanases from *F. pennivorans* (Bertoldo et al. 1999) and *Geobacillus thermoleovorans* US105 (Zouari Ayadi et al. 2008), which have a dimeric structure.

Generally speaking, highlighting the enzyme’s physico-biochemical properties is considered a crucial detrimental factor in the booklet of upcoming industrialization stage for an enzyme’s ultimate and effectual exploitation. The optimal temperature of Pull_Met is at 40 ^o^C which indicates that it is cold-adapted pullulanase type I. The literature of review has a few reports considering cold-adapted pullulanase type I with an optimal temperature between 35 and 50 ^o^C. For instance, pullulanases type I from *Paenibacillus polymyxa* Nws-pp2 (Wei et al. 2015), *Shewanella arctica* (Elleuche et al. 2015), *Exiguobacterium* sp. SH3 (Rajaei et al. 2015)d *methanolicus* PB1 (Zheng et al. 2021) exhibited an optimal temperature at 35, 35, 45, and 50 ^o^C, respectively. Likewise Pull_Met, pullulanase type I from a hot-spring metagenome (Thakur et al. 2021), B. *subtilis* BK07 (Erden-Karaoğlan et al., 2019), and *B. subtilis* PY22 (Erden-Karaoğlan et al., 2019) and *Priestia koreensis* HL12 (Prongjit et al. 2022) showed an optimal temperature at 40 ^o^C. Conversely, there is a plethora of reports addressing thermostable pullulanase type I with an optimal temperature above 50 ^o^C. The optimal temperature of thermostable pullulanases type I from *F. pennavorans Ven5* (Koch et al. 1997), *Anaerobranca gottschalkii* (Bertoldo et al. 2004), *Bacillus thermoleovorans* US105 (Messaoud et al. 2002), *Thermotoga maritima* (Kriegshäuser et al., 2000) showed an optimal temperature at 65, 70, 75, and 90 ^o^C, respectively. Pull_Met, a cold-adapted pullulanase type I, had a lower optimal temperature (40 ^o^C) than other described cold-adapted pullulanases (45–50 ^o^C) (e.g., pul-SH3 and pulPB1). As a result, Pull_Met’s advantages would be utilized in bioprocesses carried out at moderate temperatures. Currently, the usage of cold-adapted pullulanase type I in industry is much more preferable to the usage of thermostable homologues pullulanase type I from the standpoint of cost-effectiveness and energy saving.

The half-life time of the enzyme is another issue that would put constrains concerning the likely applied temperature in the bioprocesses. Enzymes with long -half life time is much preferable to homologues enzymes with short half life time in pro-longed bioprocesses. Hence, longevity in bioprocesses is another issue that would determine the choice of an enzyme with a pro-longed half life time. In this context, the Pull_Met exhibited typical characteristics of cold-adapted pullulanase type I including low-thermostability at elevated temperatures maintaining around 50 and 30% of its activity at 15–30 ^o^C after 2 h and at 45 ^o^C after 30 min, respectively. However, pullulanase type I of *Shewanella arctica* (Elleuche et al. 2015) retained 76% of its activity at 30 ^o^C after 2 h. For cold-adapted pullulanase type I of *Paenibacillus polymyxa* Nws-pp2 (Wei et al. 2015), the retained activity after 2 h was 70 and 60% after 300 min at 35 and 40 ^o^C, respectively. While, the cold-adapted pullulanase type I of *Exiguobacterium* sp. SH3 (Rajaei et al. 2015) displayed 100% retained activity after 60 min at 40 ^o^C.

The thermostability issue of a given globular protein is reportedly to be linked with the aliphatic -index which is defined as the relative volume of the protein engaged with aliphatic side chains (i.e., alanine, isoleucine, valine, and leucine). Reportedly, the aliphatic index of protein from thermophilic bacteria is higher than the ordinary proteins (Ikai 1980). The aliphatic index of the thermostable pullulanases type I from thermophilic bacteria such as *Fervidobacterium pennivorans*, *Hymenobacter mucosus*, and *Desulfurococcus mucosus* exhibited higher aliphatic indices of 86.12, 86.6, and 90, respectively compared to that of Pull_Met of 81.6. Conversely, the aliphatic indices of the thermostable *Geobacillus thermoleovorans*, *Thermococcus hydrothermalis*, and *Thermoanaerobacter thermohydrosulfuricus* were of 81.5, 81.5, and 74.42, respectively which were lower than that of Pull_Met. This would in turn reflect that the thermostability issue of a given protein is correlated with factors other than the aliphatic index. The index may be regarded as a positive factor for the increase of thermostability of globular proteins.

Concerning the optimal pH for previously reported pullulanases type I, the optimal pH spanned from 4.5 to 8.5. In this context, the Pull_Met displayed an optimal pH of 6.0 which was well- compatible with the reported range of the optimal pH for pullulanases type I. Meanwhile, pullulanase type I from *Lactococcus lactis* (Waśko et al., 2011), *Bacillus methanolicus* PB1 (Zhang et al. 2020), *Priestia koreensis* HL12 (Prongjit et al. 2022), *Bacillus megaterium* Y103 (Wu et al. 2022), and *Anaerobranca gottschalkii* (Bertoldo et al. 2004), exhibited an optimal pH of 4.5, 5.5, 6.0, 6.5, and 8, respectively. The slight acidic to near neutral pH optima for Pull_Met could be explained by the perception that the enzyme is most likely secreted internally in the cytoplasm, which displays a low pH compared to the pH in the external environment (Krulwich et al. 1997). This cellular localization of Pull_Met was additionally evidenced by the absence of a signal peptide in Pull_Met amino acid sequence as predicted by Signal IP 6.0.

The pH stability of an enzyme is an issue of a paramount importance in enzymes- dependent bioprocesses. An enzyme wide a broad range of pH stability would be more preferable than its homologues to cope well under harsh conditions. In the light of this conception, the Pull_Met showed a full stability for 5 h under a wide range of pH(s) from 2.6 to 10.0 except pH(s) 8.0 and 8.5. The pullulanase type I from *Exiguobacterium acetylicum* YH5 (Qiao et al., 2015) exhibited less stability (93%), over a wide range of pH 4–10 within 30 min, compared to that of Pull_Met. Whilst, pullulanase type I of *Paenibacillus barengoltzii* (Liu et al. 2016) demonstrated pH stability (80–100%) over a wide range of pH(s) from 5.0 to 10.5 after 30 min. A narrow-range of pH stability (6.0-8.5) as displayed by pullulanase type I from *B. megaterium* Y103 (Wu et al. 2022) with a retained activity of over 80% after 30 min.

The aptness of Pull_Met for working effectively in slightly acidic to near neutral settings in bioprocesses would be controlled mostly by its pH stability. The discrepancy in optimal pH and temperature for pullulanases type I from thermophilic, mesophilic, and psychrotolerant bacteria might be attributed to the strain difference, nature of the habitat of these bacteria, where the latter would in turn impose the mechanisms of acclimatization under harsh conditions in extremophiles habitats in terms of whole proteome with unique properties for each strain.

Reportedly, pullulanases type I of bacterial origin show metal ion preference that would promote their activity. Metal ion preference by pullulanases type I varied widely among different strains. The Pull_Met activity was not stimulated by Ca^2+^. Similar phenomenon was traced in the pullulanases from *Bacillus acidopullulyticus* (Stefanova et al. 1999) and *Anoxybacillus* sp. LM18-11(Hii et al. 2012) Despite, searching the NCBI conserved domain database (CDD) revealed that pullulanase (PbPulA) had three Ca^2+^ binding sites at D216, E224, and E245. Moreover, pullulanases type I from *Bacillus* sp. CICIM 263 (Stefanova et al. 1999) and *Thermococcus hydrothermalis* (Gantelet et al. 1998) Vent had not only been activated by Ca^2+^ but also showed enhanced thermostability. Likewise Pull_Met, the *P. polymyxa* Nws-pp2 (Wei et al. 2015) and *Thermus caldophilus* GK-24 pullulanases type I (Kim et al. 1996) were significantly stimulated by Mn^2+^, meanwhile Cu^2+^ exerted significant dramatic drop in enzyme activity. The pullulanase type I from *E. acetylicum* YH5 (Qiao et al., 2015) was significantly enhanced by Mn^2+^ and Fe^2+^ but was significantly inhibited by Cu^2+^ after 30 min pre-incubation. Likewise Pull_Met, pullulanase type I from *Lactococcus lactis* IBB 500 (Waśko et al., 2011) showed complete loss in enzyme activity after 30 min pre-incubation with Hg^2+^. Unlike Pull_Met, the pullulanase type I from *Thermus caldophilus* GK-24 (Kim et al. 1996) was stimulated significantly in presence of Ni^2+^.

Analysis of pullulan end products is an important issue in the context of determining the type of Pull_Met. The end products of pullulan hydrolysis by Pull_Met indicated its affiliation to group type I as long as the maltotriose was the sole end products of hydrolysis. This sole end product of pullulan hydrolysis along with the inability to attack α-amylose would confirm that Pull_Met was capable of attacking α,1–6 glycosidic linkage not α-1,4 glycosidic linkage. Additionally, the *in silico* sequence analysis of Pull_Met, indicating its affiliation to pullulanases type I, was in a complete agreement with the experimental results of pullulan hydrolysis end products. Likewise Pull_Met, pullulanases type I from *B. naganoensis* (Zhang et al., 2013), *Exiguobacterium* sp. SH3 (Rajaei et al. 2015) *Exiguobacterium acetylicum* YH5 (Qiao et al., 2015), *Geobacillus subterraneus* strain KCTC 3922 (Chen et al. 2022) showed only maltotriose as the end products of pullulan hydrolysis. The main end product of pullulan hydrolysis by Pull_Met, maltotriose, would reflect its likely exploitation in starch processing to produce maltotriose with a high degree of purity. Reportedly, pullulanases type I showed broad range of substrate specificity especially on polysaccharides substrates with α-1,6 glycosidic linkages such as pullulan, glycogen, amylopectin, and starch. In this context, Pull_Met obeyed this rule as it showed an activity on starch and dextrin while the ultimate activity was evidenced on pullulan.

The EDTA exhibited full dramatic loss in Pull_Met activity that would reflect that Pull_Met was a metallo-enzyme (i.e., presence of metal ions was essential for enzyme activity). Likewise Pull_Met, pullulanases type I from *E. acetylicum* YH5 (Qiao et al., 2015) and *Geobacillus kaustophilus* DSM7263 (Li et al. 2018) were metalloenzymes.

The activity of Pull_Met was stimulated by the reducing agents, β-mercaptoethanol at 10 mM. Similar results were obtained in the previous studies on pullulanases type I (Thakur et al. 2021; Wei et al. 2015). It is likely that β-mercaptoethanol would inhibit the oligomerization of the enzyme (leading to its inactivation) by breaking the disulfide bonds between monomeric subunits of the enzyme.

Reportedly, detergents imposed a great effect on stability and activity of amylolytic enzymes including pullulanases (Thakur et al. 2021). The presence of (0.1, 0.25, and 0.5 mM) of non-ionic detergents (Tween-80, Twen-20, and Triton X-100) imposed a significant enhancement in the Pull_Met activity (up to 151%) after 30 min pre-incubation (Fig. 3A) This was in accordance with previous reports describing the role of non-ionic detergents on pullulanase type I (Elleuche et al. 2015; Wu et al. 2022).

Regarding SDS, the retained Pull_Met activity was 47.11 ± 7.09 and 38.9% ± 2.02, after 30 min pre-incubation with SDS at 0.25 and 0.5 mM, respectively. In this regard, the activity of pulA from *Thermotoga neapolitana* was reported to decline to 17% in presence of 35 mM SDS (Kang et al. 2011). Moreover, the pullulanases type I from *F*. *pennavorans* Ven5 (Bertoldo et al. 1999), G. *thermoleovorans* US105 (Zouari Ayadi et al. 2008)d *arctica* (Elleuche et al. 2015) were reported to be completely inhibited in presence of 1, 3.5, and 35 mM SDS, respectively. Unlike Pull_Met, Pul-SH3 did prove to be SDS tolerant and its activity was being traced up to 350 mM of SDS (Rajaei et al. 2015).

A significant dramatic decline in Pull _Met activity up to 22.36% was noticed after 30 min pre-incubation with CTAB. Similarly, the pullulanase type I activity of *S. arctica* was lowered by increasing the concentration of CTAB (Elleuche et al. 2015).

The tolerance against detergents is a significant characteristic by which enzymes are evaluated for their potential industrial application.

At most, amylupullulanases (pullulanase type II) did show detergents stability and several studies reported the washing performance of these enzymes (Dakhmouche Djekrif et al. 2021). Conversely, a few studies did address the likely potential of pullulanase type I in the detergent industry (confined only to Pul-SH3 (Rajaei et al. 2015) and PulY103 (Wu et al. 2022)). Interestingly, in the presence of commercially detergents, Pull_Met retained the majority of its activity (around 98%) after 30 min pre-incubation with Oxi ^TM^ and Tide ™. However, around 89% of activity was retained after 30 min pre-incubation with Persil ™, Ariel ™, and Art ™. Hence, the enzyme would display a great potential in the detergent industry as a detergent additive. Among the studies that evaluated the wash performance of pullulanases (Wu et al. 2022), where pullulanase type I from *B. megaterium* Y103 exhibited the maximal R (the value of detergency) and P (rate of the value of detergency) when combined with the commercial laundry detergent BlueMoon. The pullulanase type I from *Exiguobacterium* sp. SH3 (Rajaei et al. 2015) exhibited stability of 80.4 and 93.7% in the presence of two commercial detergents, Rika (7.5% v/v) and Fadisheh (2.5% w/v), respectively.

Most of reported studies on pullulanases did highlight the stability of pullulanases type II towards organic solvents (Siroosi et al. 2014). No previous studies reported the solvent stability of pullulanases type I. Consequently, the comparison of Pull_Met stability towards organic solvents would not be justified yet. Pull_Met was stable in presence of methanol, ethanol, and butanol.

Comparing the *K*_m_ and *V*_max_ values among different enzymes is not easy task since there is a wide discrepancy in the substrates and the reaction conditions. As a rule of thumb, a low *K*_m_ value for a given enzyme does confer the high specificity of this enzyme toward the substrate and vice versa. The *k*_m_ value of pullulanase type I of microbial origin varied widely. Obviously, Pull_Met exhibited a *K*_m_ of 2.369 mg/mL, which was much smaller than those of other pullulanases type I from a hot-spring metagenome with *K*_m_ of 15.25 mg/mL (Thakur et al. 2021), *G*. *subterraneus* strain KCTC 3922 with *K*_m_ of 4.37 mg/mL (Chen et al. 2022), B. *polymyxa* Nws-pp2 with *K*_m_ of 4.0 mg/mL (Wei et al. 2015), and *Priestia koreensis* HL12 with *K*_m_ of 3.81 mg/mL. This in turn would reflect the high specificity of Pull_Met on pullulan compared to the aforementioned pullulanases type I.

Interestingly, Pull_Met exhibited potential in raw ex potato starch saccharification in synergistic co-operative action with CA-AM21 as concluded from the liberated reducing sugars in terms of glucose from the raw starch.

Typically, raw substrate hydrolysis requires the synergistic interaction of enzyme composites to achieve complete saccharification (conversion yield ≥ 80%), such as cooperation of α-amylase and pullulanase in starch hydrolysis (Pan and Lee 2005; Prongjit et al. 2022).

This indicated that pull_Met is suitable to use as the main single amylolytic enzyme, combined with other degradation enzymes, to achieve complete saccharification and could be used to develop efficient starch saccharification and modification processes.

In both the academic and industrial sectors, the vast majority of type I pullulanase has only ever been assigned to mesophilic and thermophilic homologues. Nevertheless, cold-adapted pullulanase type I is a crucial class of enzymes in bioprocessing because one can stop a reaction at low temperatures without releasing undesirable end products due to unwanted side reactions in the food industry, which are typically caused by using pullulanase type I’s thermophilic and mesophilic homologues. Pull_Met is considered one paradigm of a little bit previously reported cold-adapted pullulanase type I in the academic sector so far. In conclusion, Pull_Met is a cold-adapted type I pullulanase with added value in two commercial sectors: saccharification and detergency, despite the dearth of cold-adapted type I pullulanase.

## Electronic supplementary material

Below is the link to the electronic supplementary material.

Supplementary Material 1

Supplementary Material 2

Supplementary Material 3

Supplementary Material 4

Supplementary Material 5

Supplementary Material 6

## Data Availability

All data are available and included in the article.
