# Correction: A molecular study on recombinant pullulanase type I from *Metabacillus indicus*

**DOI:** 10.1186/s13568-023-01563-6

**Published:** 2023-06-07

**Authors:** Zahraa Z. Al-Mamoori, Amira M. Embaby, Ahmed Hussein, Hoda E. Mahmoud

**Affiliations:** Biotechnology Department, Institute of Graduate Studies and Research, Alexandria University, Alexandria, Egypt

**Correction: AMP Express (2023) 13:40** **https://doi.org/10.1186/s13568-023-01545-8**

Following publication of the original article (Al-Mamoori et al. 2023), the author noticed the errors in the textual part and in Fig. 5. These errors have now been corrected with this correction.

- The amount of reducing sugars released from saccharification of starch by the synergistic action of Pull_Met & CA-AM21 should be in terms of mg Rs/gram starch substrate instead of g RS/gram starch substrate.
- The label of Y-axis should be mg Reducing sugars/gram substrate instead of g Reducing sugars/gram substrate.

**Fig. 5:**
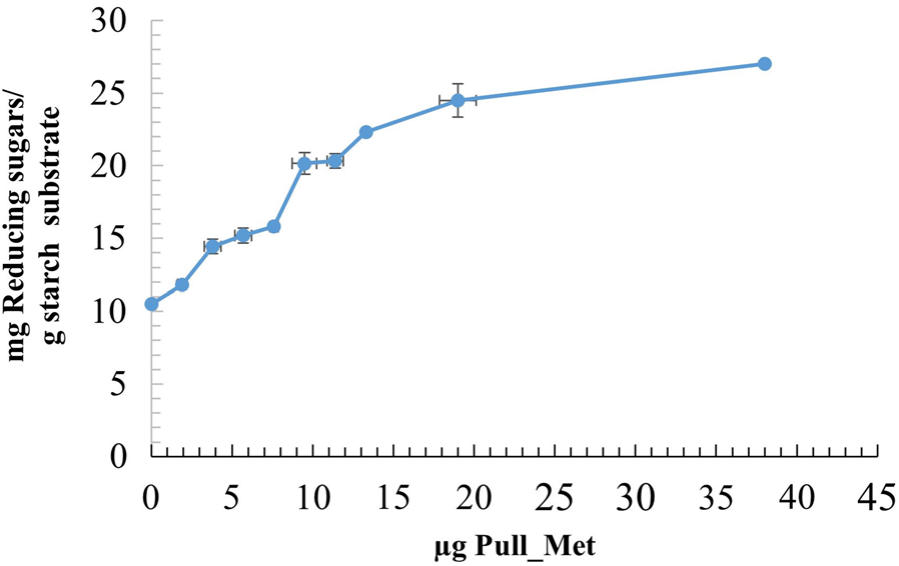
Synergistic action of gradual concentrations of Pull_Met with 21.28 U/mg CA-AM21 on raw ex potato starch saccharification. Values are the average of three readings ± SE
