# Supplementary figures and images for: A molecular study on recombinant pullulanase type I from Metabacillus indicus
Source: AMB Express. 2023 Apr 29;13:40. doi: 10.1186/s13568-023-01545-8 (PMC10148936; doi:10.1186/s13568-023-01545-8)

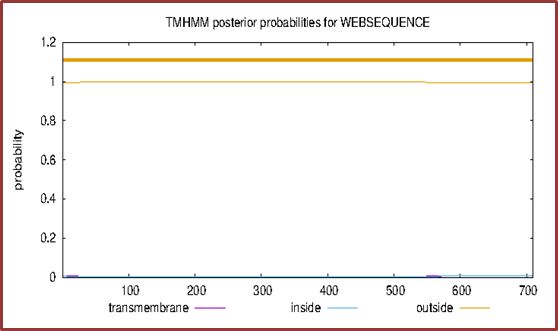

Supplement: Supplementary file 1 — Supplementary Material 1 [file 13568_2023_1545_MOESM1_ESM.jpg]

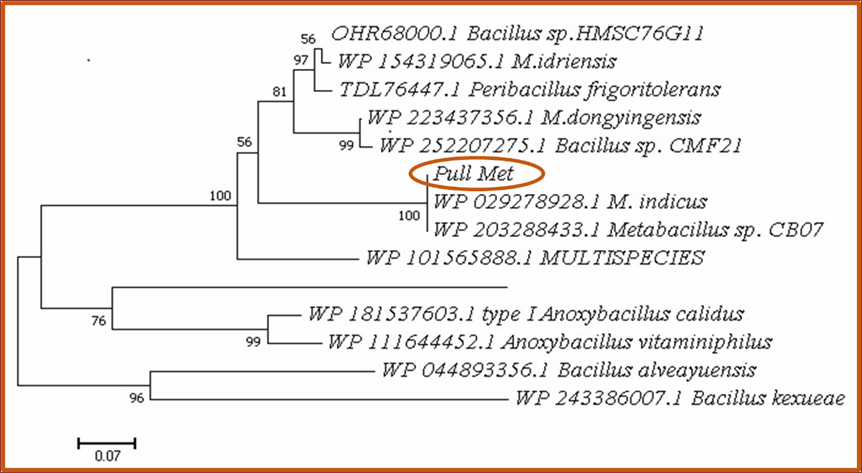

Supplement: Supplementary file 2 — Supplementary Material 2 [file 13568_2023_1545_MOESM2_ESM.tif]

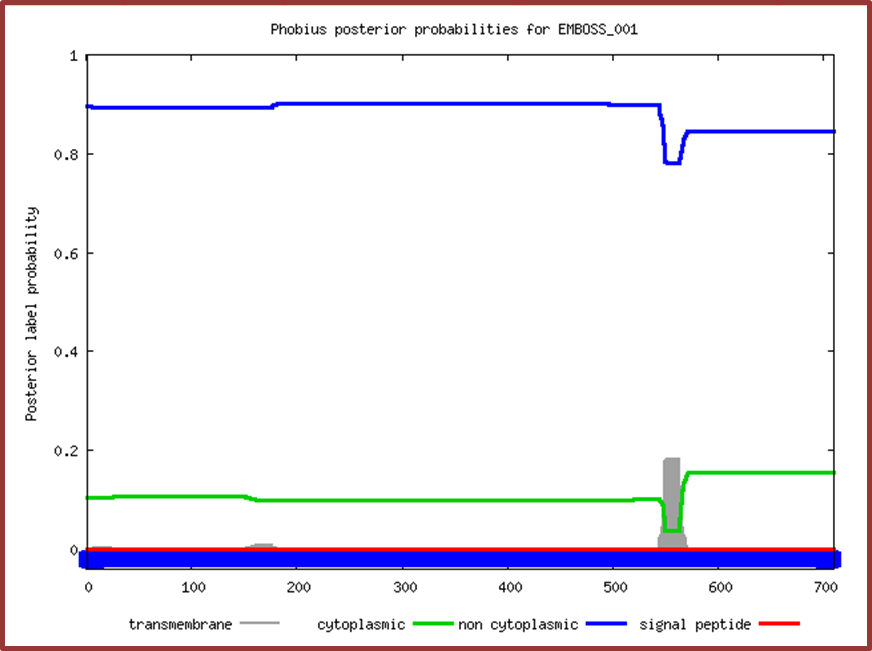

Supplement: Supplementary file 3 — Supplementary Material 3 [file 13568_2023_1545_MOESM3_ESM.tif]

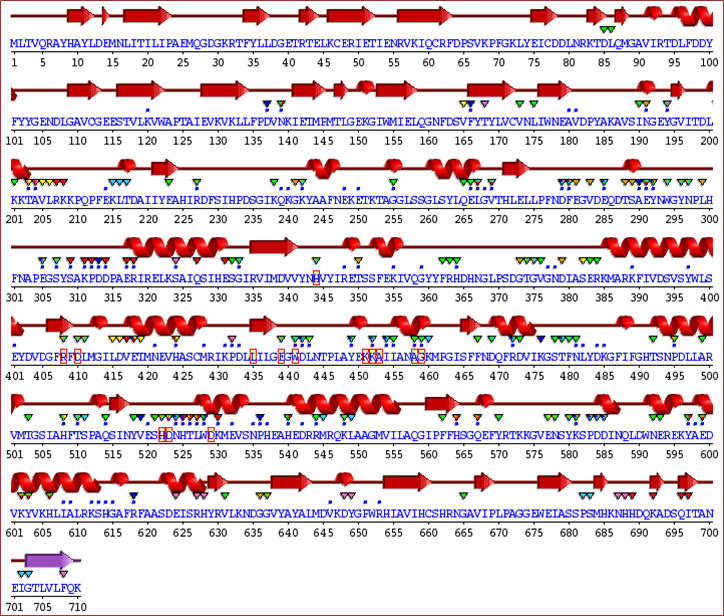

Supplement: Supplementary file 4 — Supplementary Material 4 [file 13568_2023_1545_MOESM4_ESM.tif]
