# Supplementary material for: A molecular study on recombinant pullulanase type I from Metabacillus indicus
Source: AMB Express. 2023 Apr 29;13:40. doi: 10.1186/s13568-023-01545-8 (PMC10148936; doi:10.1186/s13568-023-01545-8)
Supplement: Supplementary file 5 — Supplementary Material 5 [file 13568_2023_1545_MOESM5_ESM.docx]

**Table S1:** Sequence similarity of Pull_Met with other pullulanase protein sequences from other species

| Accession number | Species | Identity (%) |
| --- | --- | --- |
| WP_029278928.1 | *Metabacillus indicus* | 100.00 |
| WP_203288433.1 | *Metabacillus* sp. cB07 | 99.58 |
| OHR68000.1 | *Bacillus* sp. HMSC76G11 | 71.21 |
| WP_154319065.1 | *Metabacillus idriensis* | 71.21 |
| WP_223437356.1 | *Metabacillus dongyingensis* | 70.65 |
| WP_252207275.1 | *Bacillus* sp. CMF21 | 70.51 |
| TDL76447.1 | *Peribacillus frigoritolerans* | 71.21 |
| WP_101565888.1 | *Bacillaceae* | 68.12 |
| WP_044893356.1 | *Bacillus alveayuensis* | 54.52 |
| WP_243386007.1 | *Bacillus kexueae* | 54.67 |
| WP_181537603.1 | *Anoxybacillus calidus* | 56.69 |
| WP_111644452.1 | *Anoxybacillus vitaminiphilus* | 54.72 |
| MCA1032398.1 | *Bacillus timonensis* | 54.61 |
