# Supplementary material for: A molecular study on recombinant pullulanase type I from Metabacillus indicus
Source: AMB Express. 2023 Apr 29;13:40. doi: 10.1186/s13568-023-01545-8 (PMC10148936; doi:10.1186/s13568-023-01545-8)
Supplement: Supplementary file 6 — Supplementary Material 6 [file 13568_2023_1545_MOESM6_ESM.docx]

**Supplementary File:**

**Table S1:** Sequence similarity of Pull_Met with other pullulanase protein sequences from other species

**Figure S1:** Neighboring-joining phylogenetic tree constructed by MEGA7.0 deciphering the genetic relatedness between the Pull_Met and other homologous pullulanase amino acid sequences from other species. Circle represents Pull_Met. Numbers on branches represent bootstrapping values (500 re-samplings).

**Figure S2:** Output of Phobius analysis of Pull_Met amino acid sequence.

**Figure S3:** Output of TMHMM analysis of Pull_Met amino acid sequence.

**Figure S4:** Predicted secondary structure of Pull_Met, generated by SAS.
